# Development of a Mobile App Game for Practicing Lung Exercises: Feasibility Study

**DOI:** 10.2196/63512

**Published:** 2025-03-04

**Authors:** Chatkhane Pearkao, Korakot Apiratwarakul, Lerkiat Wicharit, Wiphawadee Potisopha, Arunnee Jaitieng, Sukuman Homvisetvongsa, Puthachad Namwaing, Peerapon Pudtuan

**Affiliations:** 1 Department of Adult Nursing, Faculty of Nursing, Khon Kaen University, Khon Kaen, Thailand; 2 Department of Emergency Medicine, Faculty of Medicine, Khon Kaen University, Khon Kaen, Thailand; 3 Department of Community Health Nursing and Primary Medical Care Nursing, Boromarajonani College of Nursing, Faculty of Nursing, Praboromarajchanok Institute, Ministry of Public Health, 177 Chang Phueak Road, Nai Mueang Subdistrict, Mueang Nakhon Ratchasima District, Nakhon Ratchasima, 30000, Thailand, 66 4424 3020, 66 4424 7122; 4 Deparment of Family and Community Health Nursing, Faculty of Nursing, Khon Kaen University, Khon Kaen, Thailand; 5 Department of Physical Therapy, Khon Kaen Hospital, Khon Kaen, Thailand

**Keywords:** mobile app game, practice lung exercises, feasibility study, mobile phone, pulmo device, app

## Abstract

**Background:**

Chest injuries are a leading cause of death and disability, accounting for 10% of hospital admissions and 25% of injury-related deaths. About two-thirds of patients with thoracic injuries experience complications such as blood or air in the pleural space, causing lung deflation and poor gas exchange. Proper breathing management, using tools like incentive spirometers, improves lung function and recovery. However, there is a gap in mobile-based gaming apps designed for lung exercise, which could benefit both the general population and patients recovering from lung injuries.

**Objective:**

This research aimed to develop and evaluate a mobile app game for practicing lung exercises, accompanied by a prototype device called the Pulmo device.

**Methods:**

The study involved a sample group of 110 participants from the general public. It followed a research and development methodology comprising 4 steps. The research instruments included a mobile app game, a prototype lung exercise device, and questionnaires to assess users’ satisfaction and the feasibility of both the app and the device.

**Results:**

The findings revealed that the participants demonstrated a high level of overall satisfaction with both the mobile app game and the prototype lung exercise device (mean 4.4, SD 0.4). The feasibility for the mobile app game and the prototype lung exercise device connected to the game was evaluated. The results indicated that the sample group perceived the overall feasibility to be at a high level (mean 4.4, SD 0.5).

**Conclusions:**

The research results reflected that the sample group believed the mobile app game for practicing lung exercises and the prototype device developed in this project have a high potential for practical application in promoting lung rehabilitation through gameplay. The mobile app game and the Pulmo device prototype received positive user feedback, indicating potential practical use; however, further validation is required among patients in need of pulmonary rehabilitation.

## Introduction

Chest injury is considered a leading cause of death and morbidity worldwide and is the second most common cause of death after head injuries. It is also one of the leading causes of long-term disability and hospital admissions, accounting for 10% of hospital admissions and 25% of injury-related deaths [1]. Approximately two-thirds of patients have thoracic injuries. These vary in severity from broken ribs to injuries that penetrate the heart or a torn trachea (tracheobronchial disruption). In addition, injuries resulting from concussion are the most common, with an incidence of 90% [2].

Furthermore, up to 70% of patients with thoracic injuries develop blood or air in the pleural space, preventing the lungs from fully expanding. This loss of negative pressure in the pleural space results in lung deflation and inefficient gas exchange. If correct, appropriate, and timely treatment is not received, it may cause oxygen depletion and can be life threatening [3].

Breathing exercises help improve lung function and increase quality of life. Practical breathing management often involves the use of a device known as an incentive spirometer, which helps to increase lung volume. This tool, designed for simple usage, allows patients to independently practice and improve their respiratory function. Nurses should have knowledge in breathing management. Breathing management is different from exercise. The breathing management will focus on every part of the respiratory tract, both in normal parts and in parts with pathology and should have knowledge in using the equipments in breathing management, which are used in many different types, even in the same hospital. This depends on objectives according to the doctor’s treatment plan [4].

While existing research demonstrates the effectiveness of mobile games and technology-based interventions in various rehabilitation contexts from neurological rehabilitation showing improvements in cognitive skills, motor functionality, and balance, to successful applications in pulmonary rehabilitation for chronic obstructive pulmonary disease patients with enhanced quality of life and exercise capacity, there appears to be a significant gap in developing comprehensive mobile-based gaming apps specifically designed for practicing lung exercise in the general population that could be adapted for patients recovering from lung injuries. Current studies primarily focus on either specific medical conditions like chronic obstructive pulmonary disease and stroke, or on mobile-based apps, but don’t address the potential of mobile-based gaming platforms that could serve both preventive purposes for the general population and therapeutic apps for post-lung–injury rehabilitation [5-9].

This research aimed to develop and evaluate a lung rehabilitation innovation combining a mobile-based gaming app with a prototype assessment device. The study had 2 primary objectives: first, to create an integrated system comprising a mobile application game for lung exercises paired with a prototype device for lung function assessment; and second, to evaluate this system’s potential for practical implementation by assessing both user satisfaction among the general public and its feasibility as a tool for promoting lung rehabilitation through gamified exercise.

## Methods

### Study Design

Research and development consisted of 4 steps, details are as follows:

Step 1 research established the fundamental groundwork for developing our mobile app game for practicing lung exercise. We conducted a comprehensive literature review that examined both the clinical and educational aspects of our intervention. We systematically investigated existing innovations promoting lung exercise, focusing on both traditional approaches and technological interventions. In addition, we focused on game-based learning principles and their application in health care education.

Step 2 development encompassed the systematic design and development of our mobile app game for lung exercise practice, building directly on the insights gained from our literature review in step 1.

Step 3 research marked our transition from development to real-world implementation through testing of the mobile app game with 110 healthy participants. During this testing phase, we observed how users engaged with the lung exercise game and collected valuable data regarding both user satisfaction and the application’s feasibility as a practical tool for lung exercise.

Step 4 development focused on refining both the device and app based on user feedback collected during the R2 testing phase. These improvements ensured our final product effectively addressed user needs identified during implementation.

### Development Process

**Figure 1.**
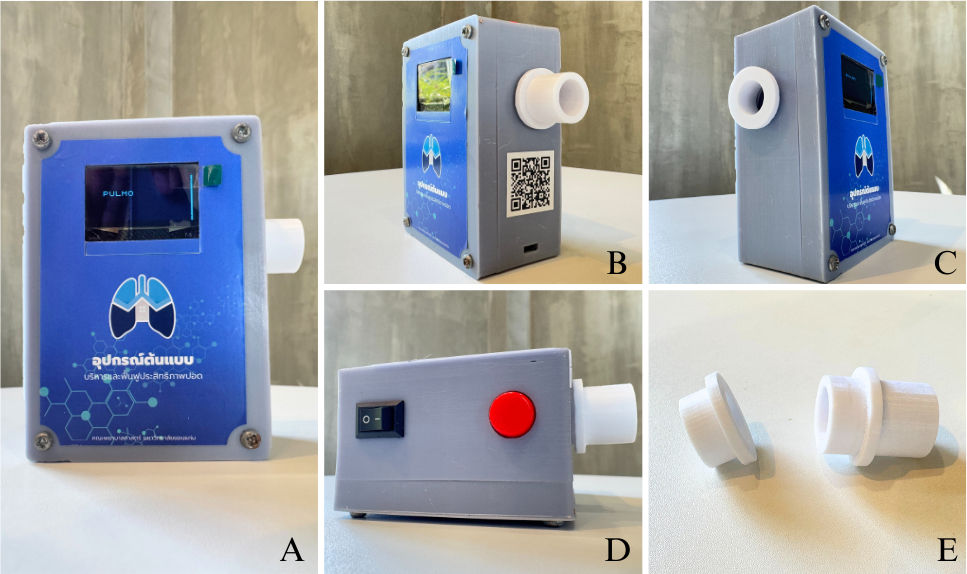
Pulmo device: (A) front view (B) lateral view (left side) (C) lateral view (right side) (D) top view (E) blowing plug (right), air outlet plug (left).

Developing an app with equipment to manage and restore efficiency is done when the air is blown, called the Pulmo device (Figure 1). The Hall effect describes the behavior of charge carriers in a material under a magnetic field. It is widely used in Hall effect sensors for accurate, noncontact detection in various fields. In the proposed lung exercise device, the sensor enables precise, real-time airflow or pressure measurements for effective monitoring [10].

The sensor receives Hall effect values, used to measure the size of the magnetic field intensity. When a magnetic field is detected in the circuit, voltage will be generated. This voltage is called Hall voltage. This voltage is responsible for detecting the magnetic flux density. These sensors basically function as linear transducers. This flow rate sensor will be connected to nodemicrocontroller unit, a board that uses ESP8266 as a processing unit.

For processing, the device will be set up as a hotspot in order to connect in a computer network manner. It is a network linking system for 2 or more devices including other types of electronic equipment that can communicate, connect, and send data between each other within the transmission control rules according to the IP, where communication protocols are used by assigning addresses. The rules for controlling transmission are determined according to the transmission control protocol or IP of each device so that receiving and sending data can easily connect to each other. After receiving the speed of the air blowing through the sensor, the data and operating status will be displayed on a 1.8-inch thin-film liquid crystal display and will prepare to send fees through the internet communication protocols used to send and receive data between the client and the server (hypertext transfer protocol) only when requesting information. The device is powered by a 3.7 volt, 800 mAh battery. The battery can be charged by a USB port and has an on or off switch for the device’s power supply circuit.

For the velocity values received from the sensors, the internal flow is analyzed. Smooth flow pattern a flow pattern in which fluid particles move in an orderly manner. There is no mixing between the fluid layers. This type of flow this generally occurs with fluids that have high viscosity and flow at low speeds or where the diameter of the pipe through which the fluid flows is very large compared with the volume of fluid flowing within the pipe. The flow characteristics of the fluid are important for its selection.

**Figure 2.**
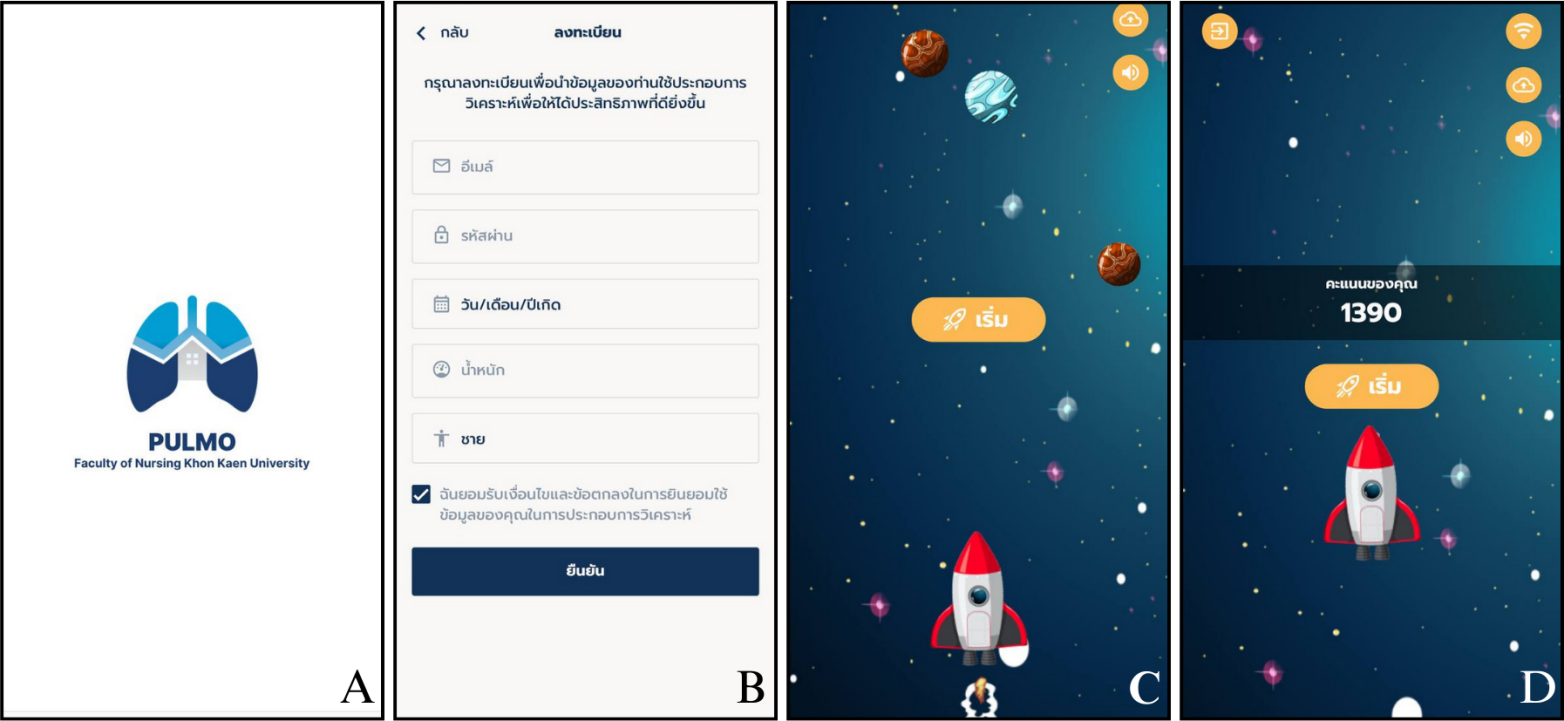
Application: (A) home page, (B) registration form, (C) game start page, (D) game end page.

The mobile app game for practicing lung exercises (Figure 2) has a total of 10 game levels, in which the number of blows to be counted as passing must be greater than or equal to 1200 L/s. There is also a chat bot function where there will be questions to choose from, and when the user selects a question, an automatic answer will pop up for the user of the device to read.

The key elements of game-based learning (rule, objective, feedback, interaction, challenge, and narrative) [11] were implemented in the Pulmo device game app. The game provides clear instructions and descriptions regarding Pulmo device connectivity and proper usage procedures (rule) and defines gamification purposes to help users practice their lung exercise (objective). The system provides real-time performance metrics during breathing exercises and immediately responds to each breath (feedback and narrative). User interaction occurs through single-player engagement with the device-connected game environment (interaction). Furthermore, this application records exercise history tracking, allowing users to benchmark against their personal bests (challenge).

### Recruitment

The study population comprised residents in Mueang Khon Kaen District, Khon Kaen Province, Thailand. The total population of 104,037 people, obtained from the Civil Registration Population Database for fiscal year 2023, was used to calculate the sample size [12]. The calculation incorporated a 95% CI, a 0.05 margin of error, and a population proportion of 0.07, based on the reported 7% prevalence of chronic obstructive pulmonary disease among Thai adults aged 40 years and above [13]. This calculation indicated a minimum required sample size of 100 participants. To account for potential incomplete data, we added a 10% buffer, resulting in a final sample size of 110 participants. Participants were selected through convenience sampling according to eligibility inclusion criteria.

Participants were recruited according to inclusion criteria. All participants were required to be healthy adults aged 18 years or older with no self-reported history of respiratory diseases, chronic lung conditions, or acute respiratory symptoms at the time of enrollment. In addition, participants needed to own a smartphone and provide informed consent for voluntary participation in the study. The study excluded individuals with pre-existing conditions that could be affected by respiratory gaming exercises, specifically those with cardiovascular disease, hypertension, hypotension, or diabetes, to ensure participant safety during the respiratory exercise protocol.

The data collection in phase R2 used 3 research instruments:

1. Demographic questionnaire collected participants’ basic information including gender, age, highest level of education, and occupation to characterize the study population.
2. Satisfaction assessment form used a researcher-developed questionnaire based on literature review and previous research [14]. This instrument comprised eight items rated on a 5-point Likert scale (5=very satisfied and 1=less satisfied). The questionnaire underwent content validity assessment by three experts, yielding a content validity index of 0.85. Internal consistency reliability testing during this study demonstrated Cronbach α coefficient of .89.
3. Feasibility assessment form evaluated users’ perspectives on implementing the mobile app games and prototype lung exercise equipment. This researcher-developed instrument, grounded in literature review and related research, contained 4 items rated on a 5-point Likert scale (5=most likely and 1=least possibility). Content validity assessment by 3 experts produced a content validity index of 0.82, and reliability testing during the study was Cronbach α coefficient of .86.

### Ethical Considerations

The researcher protected the rights of the sample subjects and received approval from the Human Research Ethics Committee, Khon Kaen University, Thailand, number HE664028, approved on February 12, 2024. After receiving approval for data collection, an experiment was conducted and data were collected, taking into account the ethics of researchers, the principle of benefit, the principle of justice, and respect for people. All participants provided written informed consent before study enrollment. The consent process included a detailed explanation of the study objectives, procedures, potential risks and benefits, data collection methods, and confidentiality measures. Participants were explicitly informed of their right to withdraw from the study at any time without any negative consequences. They were given adequate time to read the consent form, ask questions, and discuss any concerns before signing. The sample group could terminate their participation without giving reasons to the researcher, and they would not lose any benefits. All information was kept confidential and was not disclosed. The researcher analyzed the data as a whole, using it solely for educational purposes.

### Statistical Analysis

Data analysis was conducted using SPSS (version 28, IBMCorp). Demographic data were analyzed using descriptive statistics, specifically frequency and percentage distributions. Both satisfaction and feasibility scores were analyzed using means and SDs. The interpretation of satisfaction scores followed a 5-point scale: 4.51‐5 (very satisfied), 3.51‐4.5 (quite satisfied), 2.51‐3.5 (moderately satisfied), 1.51‐2.5 (less satisfied), 1.00‐1.5 (least satisfied). Similarly, feasibility scores were interpreted using the following scale: 4.51‐5 (most likely feasible), 3.51‐4.5 (highly feasible), 2.51‐3.5 (moderately feasible), 1.51‐2.5 (less feasible), 1‐1.5 (least feasible).

## Results

The study was conducted from April 25, 2024, to July 31, 2024. As shown in Table 1, 84 out of 110 participants (76.4%) were female, and 71 out of 110 participants (64.5%) were aged between 18‐39 years. In total, 56 out of 110 participants (50.9%) had a bachelor’s degree or higher education and 82 out of 110 participants (74.5%) were civil servants, government employees, or state enterprise employees.

From Table 1, this study included 84 out of 110 participants (76.4%) were female, and 71 out of 110 participants (64.5%) were aged between 18‐39 years. 56 out of 110 participants (50.9%) had a bachelor’s degree or higher education and 82 out of 110 participants (74.5%) were civil servants, government employees/state enterprise employees.

**Table 1.** Number and percentage of the sample classified by personal information (n=110).

Demographic characteristics		Participants
**Gender, n (%)**		
	Male	26 (23.6)
	Female	84 (76.4)
**Age (years), n (%)**		
	18‐39	71 (64.5)
	40‐59	36 (32.7)
	>59	3 (2.7)
**Education, n (%)**		
	Elementary school	3 (2.7)
	High school or vocational certificate	51 (46.4)
	Bachelor’s degree or higher education	56 (50.9)
**Occupation, n (%)**		
	Civil servants, government employees, or state enterprise employees	82 (74.5)
	Private employees	7 (6.4)
	Farmer	5 (4.5)
	General employee	2 (1.8)
	Student	12 (10.9)
	Unemployed or did not work	2 (1.8)

The satisfaction of users of mobile app games and prototype lung exercise equipment connected to the game is presented in Table 2. It was found that the sample group was satisfied overall at a high level (mean 4.4, SD 0.4). When consider each item. It was found that the sample group was satisfied at a high level in all items. When categorizing each item, it was found that the sample had interesting games on mobile app (mean 4.5, SD 0.6), and the strength and durability of the prototype lung exercise device is connected to the game (mean 4.5, SD 0.5). Followed by the appropriateness of the shape and size of the prototype lung exercise device is connected to the game (mean 4.4, SD 0.5).

**Table 2.** Satisfaction of users of mobile application games and prototype lung exercise equipment connected to the game.

Satisfaction topics	Mean (SD)	Result
Interesting games on mobile application.	4.5 (0.6)	High
Suitability of the game display screen on the mobile application.	4.3 (0.6)	High
Ease of use of mobile application games.	4.3 (0.5)	High
Convenience in recording basic information for using games on mobile applications.	4.3 (0.5)	High
The appropriateness of the shape and size of the prototype lung exercise device is connected to the game.	4.4 (0.5)	High
The appropriateness of the selection of materials for the prototype lung exercise device connected to the game.	4.4 (0.6)	High
The strength and durability of the prototype lung exercise device is connected to the game.	4.5 (0.5)	High
Details and clarity of the user manual.	4.4 (0.5)	High
Overall satisfaction	4.4 (0.4)	High

The feasibility of mobile app games and prototype devices that use lung exercises connected to games is shown in Table 3. It was found that the sample group was of the opinion that the overall feasibility was at a high level, (mean 4.4, SD 0.5). When considering each item, it was found that mobile app games and prototype devices that use lung exercises connected to games are useful at the highest level, (mean 4.5, SD 0.5), followed by the response of the game on the mobile app and the prototype lung exercise device connected to the game (mean 4.4, SD 0.5).

**Table 3.** Feasibility of mobile app games and prototype devices that use lung exercises connected to games.

Feasibility topics	Mean (SD)	Result
Feasibility of games on mobile applications and prototype devices that use lung exercises connected to games.	4.4 (0.5)	High
Putting a game on a mobile application and a prototype device that uses lung exercises connected to the game into practice.	4.4 (0.6)	High
Mobile application games and prototype devices that use lung exercises connected to games are useful.	4.5 (0.5)	Highest
The response of the game on the mobile application and the prototype lung exercise device connected to the game.	4.4 (0.5)	High
Overall feasibility	4.4 (0.5)	High

## Discussion

### Principal Findings

In addressing our first objective of developing an integrated lung rehabilitation innovation, our results demonstrated successful creation of a mobile-based gaming app paired with a prototype assessment device. The user satisfaction scores (mean 4.4, SD 0.4) indicate strong user acceptance of the integrated system, particularly in terms of interesting mobile app games and the strength and durability of the prototype lung exercise device connected to the game. These findings align with previous research [15] in which the researchers similarly found that gamified respiratory interventions achieved high user acceptance rates in their 2021 study.

Analysis of satisfaction data revealed important insights about our integrated system’s design and functionality. Participants expressed high satisfaction with 2 key aspects including the mobile app game’s visual appeal and the durability of the prototype lung exercise device. The physical design elements, specifically the shape and size of the prototype device, also received favorable ratings. These findings align with research emphasizing the importance of aesthetic appeal and ergonomic design in health care devices, particularly for maintaining user engagement in rehabilitation tools. However, our assessment also identified an area requiring improvement such as the process of recording basic information within the mobile app. Similar challenges in user data management have been noted in other digital health interventions, such as in the study by Ezeonu et al [16].

Regarding our second objective of evaluating implementation potential, the feasibility assessment results (mean 4.4, SD 0.5) suggest that our system shows promise for practical application in lung rehabilitation. This finding particularly resonates with Rung et al’s [17] work, which demonstrated that game-based mindfulness interventions can effectively promote consistent practice adherence. User feedback analysis revealed high satisfaction ratings for the “Headspace app,” with participants reporting positive acceptance and engagement with the meditation platform.

The feasibility assessment revealed strong potential for practical implementation of our integrated system. Participants rated the utility of the mobile app game and connected prototype device at the highest level of feasibility, suggesting robust practical value for lung rehabilitation purposes. The system’s responsiveness, a critical factor in gaming interventions, also received high feasibility ratings. These positive evaluations of both use and responsiveness indicate that our integrated approach successfully addresses key requirements for real-world implementation in lung rehabilitation practice. These findings align with current trends in digital health interventions, where responsive, user-friendly systems have been shown to enhance engagement with therapeutic exercises [16].

The high feasibility ratings for both use and responsiveness support previous research indicating that gamified approaches can effectively bridge the gap between traditional rehabilitation exercises and patient engagement. Our results particularly complement studies [18], in which researchers found, in their 2024 systematic review, that interactive digital platforms incorporating augmented reality for physical exercise show significant promise for widespread adoption, with demonstrated improvements in both physical activity levels and mental health outcomes across diverse populations [18]. A previous study [19] demonstrated that mobile apps with visual elements significantly outperform traditional audio-only approaches for breathing training. Their study proved that wave-based visualizations led to both measurably deeper breathing and higher user satisfaction, establishing that well-designed visual interfaces enhance respiratory exercise outcomes [19].

The positive satisfaction ratings for both physical and digital components validate our integrated approach to lung rehabilitation, while the identified area for improvement offers a clear pathway for system enhancement. The strong feasibility scores across both technical performance and practical use suggest that our integrated system has significant potential for further development and implementation in clinical settings.

### Limitations and Future Research

This study developed a mobile app game for practicing lung exercises, but it was tested only on healthy volunteers, limiting its applicability to patients requiring lung rehabilitation. The short testing period also restricted the assessment of long-term effectiveness. Future research should involve clinical trials with patients, such as postoperative or chest trauma patients, to evaluate the application’s impact on lung function and its role in pulmonary rehabilitation.

### Conclusions

This study developed a mobile app game for practicing lung exercises, accompanied by a prototype device called the Pulmo device, which connects to the app. The innovation was used by the general public and user feedback indicated high levels of satisfaction and suggested the potential for practical app. However, future studies should evaluate the effectiveness of this innovation in patients requiring pulmonary rehabilitation to further validate its clinical benefits.

## References

[1] AlSulaiman RS, Al Abbas SM, Alshaikh ZA (2023). Causes and pattern of chest trauma among adults: a scoping review of studies from the middle east. Cureus.

[2] Ludwig C, Koryllos A (2017). Management of chest trauma. J Thorac Dis.

[3] Sriprom K, Samartkit N, Masingboon K (2020). Factors related to lung rehabilitation behavior in chest trauma patients with intercostal chest drainage [Article in Thai]. J Fac Nurs Burapha Univ.

[4] Rattanakanlaya K (2018). Coaching on breathing exercise by applying incentive spirometer in post-operative patients [Article in Thai]. J Royal Thai Army Nurses.

[5] Sagary R, Malim N, Abdullah NL, Mohamad W, Ahmad AL (2023). Impact of mobile games-aided neurorehabilitation: a systematic literature review. Malays J Med Sci.

[6] Wang YQ, Liu X, Ma RC (2020). Active video games as an adjunct to pulmonary rehabilitation of patients with chronic obstructive pulmonary disease: a systematic review and meta-analysis. Am J Phys Med Rehabil.

[7] Chung C, Lee JW, Lee SW, Jo MW (2024). Clinical efficacy of mobile app-based, self-directed pulmonary rehabilitation for patients with chronic obstructive pulmonary disease: systematic review and meta-analysis. JMIR Mhealth Uhealth.

[8] Joo S, Lee K, Song C (2018). A comparative study of smartphone game with spirometry for pulmonary function assessment in stroke patients. Biomed Res Int.

[9] Abdou FA, Abd Elbaky MM, Ahmed NA (2023). Effect of using mobile games on patients with acute stroke during cognitive rehabilitation at the intensive care unit. Alexandria Scientific Nursing Journal.

[10] Hall EH (1884). The Hall effect. Science.

[11] Yunus E, Zaibon SB (2021). Connecting computational thinking (CT) concept with the game-based learning (GBL) elements. Int J Interact Mob Technol.

[12] Daniel WW (2010). Biostatistics: Basic Concepts and Methodology for the Health Sciences.

[13] Thai Health Promotion Foundation (2019). Reveals the lung disease situation is still worrying [Article in Thai].

[14] Laha W, Hiruntrakul A, Ninprapan A (2018). The satisfaction of user in the isometric leg strength dynamometer in sitting position for field test [Article in Thai]. KKU Res J.

[15] Simmich J, Mandrusiak A, Smith ST, Hartley N, Russell TG (2021). A co-designed active video game for physical activity promotion in people with chronic obstructive pulmonary disease: pilot trial. JMIR Serious Games.

[16] Ezeonu NA, Hertelendy AJ, Adu MK (2024). Mobile apps to support mental health response in natural disasters: scoping review. J Med Internet Res.

[17] Rung AL, Oral E, Berghammer L, Peters ES (2020). Feasibility and acceptability of a mobile mindfulness meditation intervention among women: intervention study. JMIR Mhealth Uhealth.

[18] Piqueras-Sola B, Cortés-Martín J, Rodríguez-Blanque R (2024). Systematic review on the impact of mobile applications with augmented reality to improve health. Bioengineering (Basel).

[19] Chittaro L, Sioni R (2014). Evaluating mobile apps for breathing training: the effectiveness of visualization. Comput Human Behav.

